# Prothioconazole Stress Reduces Bacterial Richness and Alters Enzyme Activity in Soybean Rhizosphere

**DOI:** 10.3390/toxics12100692

**Published:** 2024-09-25

**Authors:** Ronggang Zhai, Mengchen Shi, Panpan Chen, Yi Wang

**Affiliations:** 1 Department of Plant and Environmental Health, School of Resources and Environment, Anhui Agricultural University, Hefei 230036, China; 2 College of Electronics and Information Engineering, Anhui Post and Telecommunication College, Hefei 230031, China

**Keywords:** prothioconazole, enzyme activity, microbial community, rhizosphere soil

## Abstract

Prothioconazole (PTC) is currently a popular triazole fungicide. In recent years, as the use of PTC has increased, there has been growing concern about its environmental and toxicological effects. Here, we studied the effect of PTC on the growth of soybean plants and further analyzed the enzyme activity and microbial community of rhizosphere soil after PTC treatment through 16S rRNA gene high-throughput sequencing and fungal ITS. Changes in structural diversity and species richness were measured using Simpson’s diversity index, Shannon’s diversity index and the Chao1 and ACE algorithms. The statistical *t*-test was applied to test whether the index values were significantly different between the two groups. The results showed that the contents of malondialdehyde (MDA) and H_2_O_2_ increased after the recommended dose of PTC, indicating that PTC has a strong toxic effect on plant growth, thus affecting the healthy growth of plants. In the presence of PTC, the species richness of fungi and bacteria decreased in all three soil types (black soil, yellow earth and red earth), and the community structure also changed significantly (the *p*-values were all less than 0.05). *Proteobacteria*, *Actinomycetota*, *Bacteroidota* and *Acidobacteriota* were the main bacteria, and the abundance of *Acidobacteriota* and *Chloroflexi* increased. The dominant fungal communities were *Ascomycota* and *Mortierellomycota*. The increased abundance of potentially beneficial microorganisms, such as *Sphingomonadaceae*, suggested that plants may be resistant to PTC stress by recruiting beneficial microorganisms. PICRUSt analysis showed that the metabolism-related functions and membrane transport pathway of rhizosphere bacterial community were inhibited after PTC stress. Spearman correlation analysis revealed a weak correlation between key fungal taxa and rhizosphere variables in the presence of PTC. Therefore, compared with those in the fungal community, the bacterial community was more likely to help plants resist PTC stress, indicating that these key fungal groups may indirectly help soybean growth under PTC stress by affecting the bacterial community.

## 1. Introduction

The rational use of pesticides is an the important means of agricultural production that can effectively control the effects of diseases damage, pests and grasses and improve the yield and quality of crops [1,2]. However, with the increasing use of pesticides, some resistant fungal mutants have developed through the mutation of the target of pesticides [3]. Pesticides have developed a moderate resistance risk. Prothioconazole (PTC) is a new broad-spectrum triazole fungicide developed by Bayer, and it is the third largest product among global fungicides, belonging to the triazole family of sterol 14α-demethylation inhibitors (DMIs). The mechanism of action is to inhibit the activity of C-14 demethylase in the sterol biosynthesis process, thus destroying the formation of the cell membrane of pathogenic fungi, so as to achieve the purpose of fungicide [4]. Meanwhile, studies on the potential causes of resistance have identified three main mechanisms of resistance to DMI fungicides: (i) alterations in the amino acid sequence of the CYP51 protein leading to a reduced affinity for azoles [5]; (ii) alterations in the upstream promoter region leading to CYP51 gene overexpression [6]; and (iii) overexpression of efflux pumps, including the atp-binding cassette (ABC) transporter and the major facilitator superfamily (MSF) transporter of the trans transporter, which reduces susceptibility by effluxing the fungal cells. And, it has been shown that prothioconazole treatment (0.1 µg/mL) resulted in altered expression of all target genes of FgCYP51, as well as prothioconazole-resistant mutants compared to wild-type parental isolates [7]. PTC not only has excellent protection [8], treatment and eradication activities [9], but also has a long duration. However, PTC is easily degraded, and the main metabolite is thioketozole [10]. However, the impact of thioketozole and PTC residues on the soil environment cannot be ignored.

The soil pollution caused by improper use of pesticides is becoming increasingly serious. The importance of soil is self-evident. Soil health (also referred to as soil quality) is closely related to microbial community structure, enzyme activity and soil type [11]. Soil health is directly related to plant growth, because healthy soil can support plant growth and withstand changes in environmental conditions. In addition, maintaining soil fertility and productivity through a range of biological processes via soil microbes is considered a key strategy for sustainable agricultural development [12]. The characteristics that ensure soil health depend on soil type, soil microbial diversity and soil biological activity.

Soil microorganisms mainly carry out oxidation, ammoniation, nitrogen fixation, vulcanization and nitrification processes in soil [13] and play a role in promoting the decomposition of soil organic matter and nutrient conversion [14]. Rhizospheric microorganisms play an important role in ecosystems, and the effects of pesticides on microorganisms are complex and diverse [1,15]. In recent years, many studies have focused on the effects of herbicides on soil microorganisms. For example, it has been reported that high concentrations of atrazine stimulate an increase in soil bacteria [16]; dichloroquinolinic acid increases the number of aerobic bacteria in rice soils within a short period of time [17]; and the number of soil fungi is strongly suppressed by the continuous application of methamphetamine [18]. In addition, a study found that the recommended doses of four herbicides (cinosulfuron, prosulfuron, thifensulfuron methyl, triasulfuron) had a low effect on the soil flora, while herbicides at 10 times the recommended dose were toxic to the soil [19]. The use of pesticides may also affect crop yields. Luo et al. found that trichlorfon and carbosulfan, which are frequently applied to fruit trees, inhibited the activity of polygalacturonase (used for enzymatic hydrolysis of fruit juice to increase the yield) and reduced the yield of apple juice [20]. Most of the chemicals applied in farmlands are scattered into the soil and can subsequently impact soil microorganisms [21]. Therefore, the effect of chemical agents on rhizospheric microorganisms after application in farmlands has become an important index for evaluating the safety of these agents, and is also a hot topic in pesticide ecotoxicology research [22].

Soil enzymes are important indicators of soil fertility and are the driving force of soil metabolism [12], they participate in various biochemical processes in soil, and their activities reflect the relative intensity of biochemical processes under certain soil ecological conditions. There are many studies on the influence of soil enzyme activity being conducted worldwide [23,24,25]. The enzymes studied included catalase, sucrase, and urease. The effects of triazole fungicides on enzymes in soil have been evaluated, usually based on measurements of the activities of several enzymes in the presence of pesticide dehydrogenase (DHA), urease (UA), phosphatase (PHA) and protease (PA) [26]. For example, it has been shown that dehydrogenase is most sensitive to the use of the triazole fungicide Difenoconazole. When the fungicide was applied at 0.150 mg/g soil, DHA activity decreased by 90.16% after 21 days under controlled laboratory conditions. Similarly, the highest concentration (150 mg/kg) of Difenoconazole resulted in a decrease in the activities of UA, PHA, and PA under laboratory conditions [27]. Therefore, the effects of pesticide residues on soil health can also be reflected by soil enzyme activity.

The problem of pesticide residue has received increasing attention. In previous scientific research, toxicological research and environmental risk assessments of pesticides were based on individual information, and few studies have evaluated the rhizosphere microbiome, especially the relationship between the rhizosphere microbiome and plants under fungicide stress. Hence, in this study, we comprehensively evaluated the environmental ecological risks of fungicides by exploring the changes in the inter-root soil microbial and enzyme activities of soybeans under PTC stress and their effects on soybean growth, with a view to contributing to the elucidation of the interactions between plants and microorganisms. Our findings provide data support for environmental risk assessments of fungicides.

## 2. Materials and Methods

### 2.1. Soil Characterization

Soybeans are commonly grown throughout China and are produced in the northeast, north China, Shaanxi, Sichuan and the lower reaches of the Yangtze River. Therefore, representative black soils in Heilongjiang Province, yellow loam soils in Henan Province and red loam soils in Jiangxi Province were selected to investigate the effects of PTC on soybean inter-root microorganisms and soil enzyme activities in soil. This will help us to further understand the toxic effects of PTC on soil and provide a theoretical basis for the rational application of PTC. The vegetable soil in three different areas was selected as the test soil, and no pesticides had been used in the last five years. The test soil was taken from garden plots that had not been treated with pesticides in the last five years in Harbin, Heilongjiang Province (45°8′ N, 126°51′ E), Zhengzhou, Henan Province (35°76′ N, 115°04′ E) and Nanchang, Jiangxi Province (28°76′ N, 115°83′ E). The physical and chemical properties of the soil in the three locations are shown in Table 1.

### 2.2. Pot Experiment

The research was conducted in a greenhouse on the roof of a biotechnology building at Anhui Agricultural University (31°24′18.97″ N, 121°29′21.88″ E) in China. After grinding and sifting the soil, it was mixed with 20 mg/L PTC solution and set aside. In this experiment, the soil in the three areas was divided into two treatments: (a) treated soil and (b) untreated soil. Each treatment was repeated six times. The treated soil was mixed with 10 mL of 20 mg/L PTC solution, and the untreated soil was mixed with 10 mL of dimethyl sulfoxide solution as a control. The soybeans (*Glycine max* (L.) Merr. ‘Zhonghuang 13′) seeds with 30% (*v*/*v*) H_2_O_2_ were sterilized before sowing [28]. Then, the plants were washed thoroughly 5 times with deionized water, and evenly sized seeds were selected for planting in pots filled with autoclave potting media [29]. When three pairs of leaves were growing, the plants with the same growth were selected and transplanted into a plastic pot (3.5 L). Irrigation was performed daily using a gravimetric method based on the soil water retention capacity (WHC) to maintain soil water at 70% of the field capacity. The experimental conditions were carried out at 25 °C, 80% relative humidity and 375 μmol photons/(m^2^s). The samples were analyzed after 7, 10 and 14 days of stress.

### 2.3. Plant Harvesting, Soil Sampling and Property Analysis

The samples were divided into plant material (leaves), rhizosphere soil and bulk soil. The leaves of all the plants were cut and mixed together before being fully ground in a frozen grinder at 4 °C, immediately placed in a sterile centrifuge tube and stored at −20 °C in a refrigerator until use to detect the oxidative stress and antioxidant kinase activity of the soybean leaves. The residual soil (approximately 1–3 mm thick) on the root surface of the soybean plants was considered to be the rhizosphere [30]. The plants were carefully removed from the soil, the loose soil was shaken off, and the rhizosphere soil was gently removed. The samples were immediately frozen in a −80 °C freezer for further microbial analysis. The other part of the soil was screened and stored at 4 °C for the detection of soil enzyme activity. The third part was air-dried at room temperature for the detection of soil chemical properties. The contents of MDA and H_2_O_2_ were determined to determine the degree of oxidative damage [31]. The contents of H_2_O_2_ and MDA were determined using a test kit provided by Beijing Boxbio Science & Technology Co., Ltd, Beijing, China. Superoxide dismutase (SOD) and peroxidase (POD) activities were detected using enzyme-specific commercial kits obtained from Beijing Boxbio Science & Technology Co., Ltd. [32]. An SOC analyzer was used to determine the soil organic carbon (SOC) content via the combustion oxidation nondispersive infrared absorption method (Multi N/C 3100, Analytik, Jena, Germany). The soil pH was measured using a pH meter (ST2100, Ohaus, Changzhou, China). Soil total phosphorus (TP) was extracted with H_2_SO_4_-HClO_4_ and determined using the molybdenum blue method (710 nm) via an ultraviolet spectrophotometer (UV-1900i, Shimadzu, Kyoto, Japan). The soil total nitrogen (TN) concentration was determined via the Kjeldahl method [33].

### 2.4. DNA Extraction

We took 250 mg of rhizosphere soil to use for DNA extraction. The total DNA in the soil samples was extracted using a soil DNA extraction kit purchased from Chengdu Fuji Biotechnology Co., Ltd. (Chengdu, China), to exclude or minimize the effect of non-specific bands and non-specific amplification product bands and to select single electrophoretic bands and non-contaminated DNA for subsequent detection. This was carried out to ensure that the size and number of target fragments could be assessed more accurately, thus ensuring the reliability and validity of the experimental results.

### 2.5. PCR Amplification and Sequencing of Bacterial 16S rRNA Gene and Fungal ITS

For this study, Shengong Bioengineering (Shanghai, China) Co., Ltd., was commissioned to conduct high-throughput sequencing of bacterial 16S rRNA (V3-V4 region) and fungal ITS region (ITS1-ITS2 region). The V3-V4 (variable region) region of the 16S rRNA gene was amplified using 314F (CCTACGGGNGGCWGCAG) and 805R (GACTACHVGGGTATCTAATCC) priors. The ITS1-ITS2 (variable region) region of ITS region was amplified using ITS1F (GCTGCGTTCTTCATCGATGC) and ITS2 (GCTGCGTTCTTCATCGATGC) primers. The PCR products were sequenced on the Illumina MiSeq platform. Double-end sequencing was prepared by constructing paired-end libraries. Sequencing primer binding sites were added to both ends of the splice when constructing the DNA library to be tested. After the first round of sequencing was completed, the template strand of the first round of sequencing was removed, and the Paired-End Module was used to guide the regeneration and amplification of the complementary strand at the original position to achieve the amount of template used for the second round of sequencing. The second round of complementary strand synthesis sequencing was then performed. We used the V600 cycle kit. Fastp V0.23.4 software was used for quality control of the double-ended original sequencing sequences, and FLASH software was used for concatenation. UPARSE software was used to perform OTUs clustering on the concatenated sequences according to the similarity of 97% of the sequences and to eliminate chimeras. Operational taxonomic units (OTUs) were determined using the UNITE reference database (http://unite.ut.ee/index.php, accessed on 1 November 2023) for ITS and the SILVA reference database (http://www.arb-silva.de (accessed on 2 September 2024)) for 16S rRNA genes as previously described. Alpha diversity was calculated using the Simpson’s and Shannon–Wiener diversity indices by applying the ‘diversity’ function (Vegan package). The relative abundance of microbes was determined as a percentage.

### 2.6. Statistical Analysis

The contents of MDA and H_2_O_2_ in the soybean leaves and the activities of antioxidant enzymes and soil enzymes were analyzed and their significance was tested (SPSS 19.0). The changes in alpha diversity, species abundance and community structure of rhizosphere flora were analyzed (Prism 9.5). Spearman correlation analysis was used to analyze the correlation between the soil microbial community, enzyme activity, and soil treatments (Origin 2023). Principal component analysis (PCA) was used to analyze changes in the soil microbial communities. PICRUSt analysis was used to analyze the changes in rhizosphere bacterial community function (PICRUSt2). A *p*-value of less than 0.05 was considered to indicate statistical significance. If necessary, the original data were normalized or logarithmically transformed before analysis. Normalization was performed prior to PCA. This was carried out to help to improve the numerical stability of the algorithm and avoid computational errors due to too large or too small a range of data.

## 3. Results and Discussion

### 3.1. Effects of PTC on the MDA Content, H_2_O_2_ Content and Antioxidant Enzyme Activity

**Figure 1:**
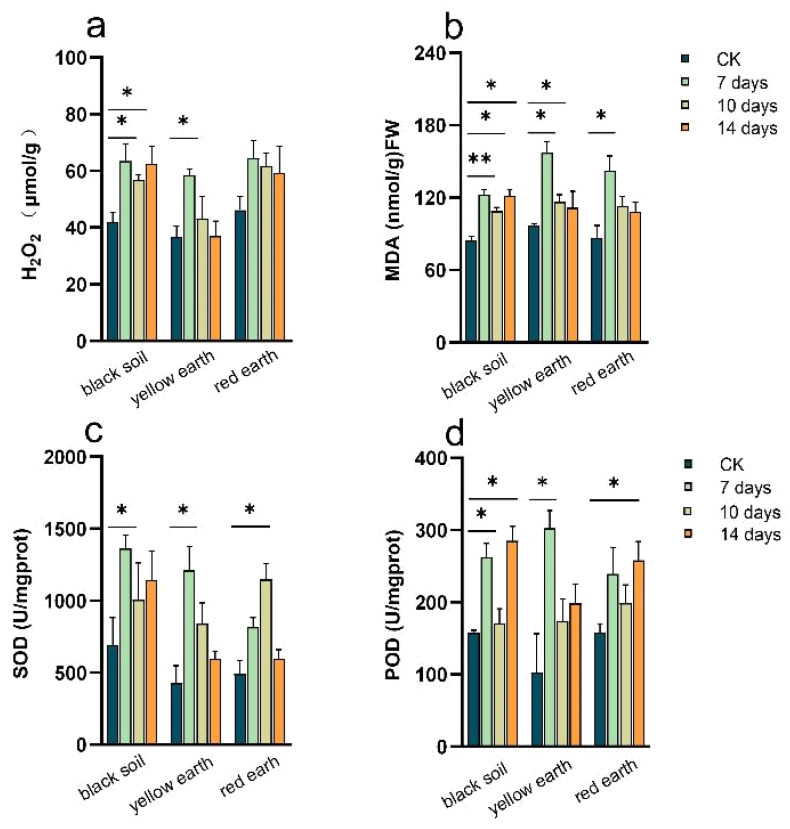
Changes in antioxidant enzyme activities, MDA content and H_2_O_2_ content after 7, 10 and 14 days of PTC treatment. (**a**) H_2_O_2_; (**b**) MDA; (**c**) SOD; (**d**) POD (*p* < 0.05). (MDA: malondialdehyde; SOD: superoxide dismutase; POD: peroxidase. * indicates a significant difference between the two sets of data. ** indicates a very significant difference between the two sets of data).

The effects of PTC treatment on the soybean defense system were investigated by measuring the activities of H_2_O_2_ and MDA in the soybean leaves (Figure 1). After 7 days of PTC stress, the MDA content of the three soils significantly increased by one time compared with that of the control. After 10 days of PTC stress, there were still significant changes in the black soil and yellow earth. At 14 days, the level of MDA was greater than that in the control group in the three soils, but the difference was not significant (Figure 1a). After 7 days of PTC treatment, the H_2_O_2_ content increased significantly. It decreased slightly but was still greater than that in the control group after PTC treatment for 10 and 14 days. The H_2_O_2_ level in the soybean plants planted in black soil exhibited the most obvious change, increasing by 1.52- and 1.35-fold after 7 and 10 days of stress, respectively, which were significantly different from those of the control group; it increased slightly in the red earth, but there was no obvious change in weight in this group (Figure 1b). The SOD and POD activities increased after PTC stress. The SOD activity changed most prominently after 7 days of stress, and its activity in black soil, yellow earth and red earth was 1.96-, 2.83- and 1.66-fold greater than that in the control group, respectively (Figure 1c). After 10 days of PTC stress, the POD activity in the black soil and red earth soils was still high and was 1.08- and 1.26-fold greater than that in control group, respectively (Figure 1d).

MDA and H_2_O_2_ are indicators of lipid peroxidation (LPO), and changes in their contents can reflect the balance of reactive oxygen species in plants. An increased H_2_O_2_ content may indicate oxidative stress (OS) in plants [1]. The MDA content of the soybean plants treated with PTC significantly increased, which showed that PTC induced oxidative stress and damaged the cell structure. The decrease in MDA content in the late stage of the experiment indicated that the plant damage symptoms had been alleviated, which might be attributed to the protective mechanism of soybean itself, such as the increase in related protective enzymes, which slowed down the degree of membrane peroxidation and reduced the membrane permeability, and at the same time effectively reduced the malondialdehyde content. SOD and POD can detoxify H_2_O_2_ and other reactive oxygen species (ROS) caused by pesticides or other pollutants [2,29]. The apparent increase in SOD and POD activity suggested that soybean plants have evolved an antioxidant enzyme system that can convert ROS into nontoxic compounds [24]. These results indicated that the antioxidant system of soybean plants was activated after PTC stress to prevent oxidative damage caused by PTC stress.

### 3.2. Effect of PTC on Soil Enzyme Activity

**Figure 2:**
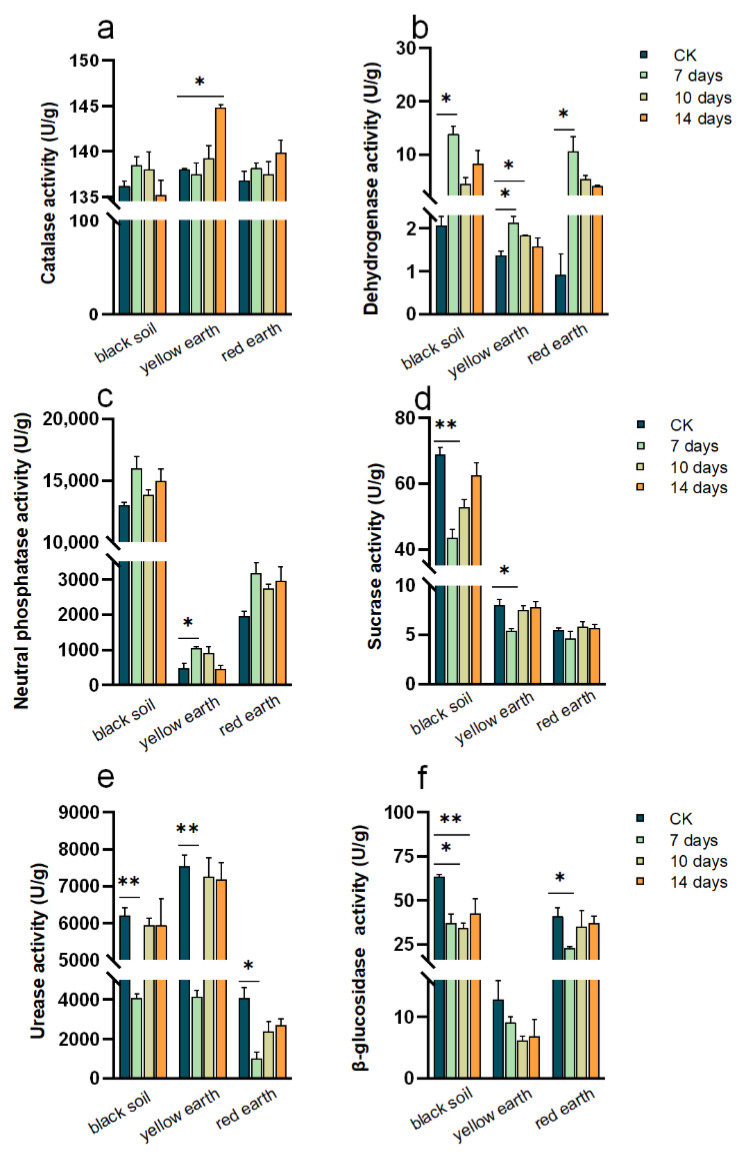
Effects of prothioconazole on essential soil enzymes ((**a**): catalase; (**b**): dehydrogenase; (**c**): neutral phosphatase; (**d**): sucrase; (**e**): urease; (**f**): β-glucosidase) after its application on soybean plants (*p* < 0.05) (* indicates a significant difference between the two sets of data. ** indicates a very significant difference between the two sets of data).

Soil catalase (CAT) activity showed an overall increasing trend after the application of PTC, and CAT activity increased significantly after 14 days of application on yellow earth (Figure 2a). After 7 days of PTC stress, the activity of dehydrogenase (DHA) in these three kinds of soil increased greatly, and after 10 and 14 days of stress, the activity of soil DHA slightly decreased (Figure 2b). There was no significant change after 7 days, 10 days or 14 days of stress in the black soil or red earth, but the neutral phosphatase (NP) activity in the yellow earth clearly increased after 7 days of stress (Figure 2c). The activity of sucrase (SC) in the three soils decreased significantly after 7 days of PTC stress, but increased at 10 and 14 days, and then tended to stabilize (Figure 2d). The activity of urease (UE) and β-glucosidase enzymes (β-GC) in black soil changed sharply, especially after 7 days of stress. The activity of UE in yellow earth decreased significantly after 7 days of stress, increased after 10 days and 14 days, but the activity of UE in red earth did not change significantly. Overall, the β-GC activity in the yellow earth showed a decreasing trend, and the β-GC activity in the red earth decreased significantly after 7 days of stress and gradually stabilized after 10 days and 14 days (Figure 2f). Differences in soil enzyme activities may be related to soil microorganisms and the physicochemical properties of soil microorganisms. Black, loess and red soils differ in texture, moisture status, structure and temperature, and thus have a direct effect on soil enzyme activity. Bacteria, fungi and actinomycetes are the main sources of soil enzymes. Certain bacteria in the soil between roots secrete laccase, while fungi and actinomycetes release specific enzymes into the soil that are closely related to the cycling of nutrients such as carbon, nitrogen and phosphorus in the soil. The results showed that PTC stress affects the enzyme activity of soybean rhizosphere soil, which may affect the nutrient supply to plants and lead to plant growth. This is consistent with the reported conclusion that interference in enzyme activity by fungicides may have deleterious effects on microbial communities and their activities [19]. Even though pesticide applications at recommended doses may cause minor and transient changes in the populations or activities of soil microorganisms, it is clear that repeated pesticide applications over a long period of time are known to interfere with biochemical equilibria, thus reducing soil fertility and productivity by affecting localized metabolism and enzyme activities [23].

### 3.3. Effects of PTC on the Rhizosphere Microbial Community

High-throughput Illumina sequencing revealed a total of 6,953,869 original reads from all 36 rhizosphere soil samples, with the number of reads per sample ranging from 42,617 to 128,516. A phylogenetic classification analysis indicated that these rhizosphere bacterial sequences were within 13 phyla and 53 families. Phylogenetic analysis revealed that these rhizosphere fungal sequences belonged to 12 phyla of 51 families.

**Figure 3:**
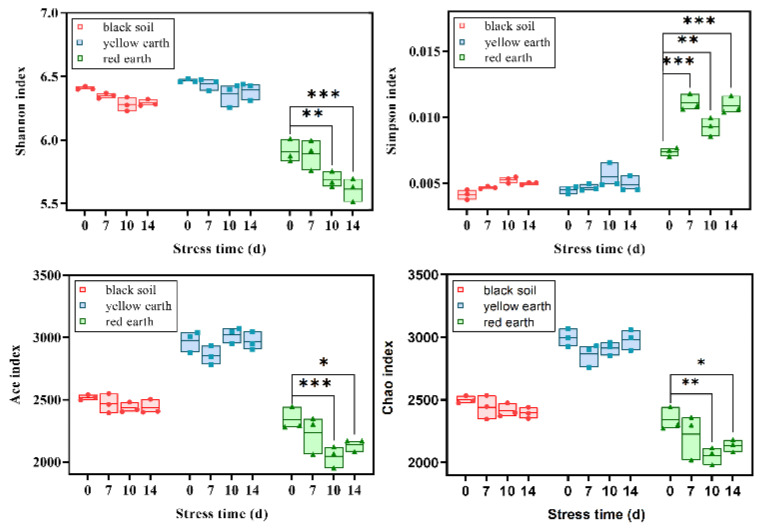
Changes in the diversity indices (Simpson, Shannon) and richness indices (Chao1, Ace) for the soybean rhizosphere bacterial community after 7, 10 and 14 days of PTC treatment (* means that the p value is less than 0.05, ** means that the p value is less than 0.01, and *** means that the p value is less than 0.001).

The species richness and diversity were compared based on four indices (the Simpson, Shannon, Chao1 and Ace indices). After PTC treatment, the Simpson and Shannon indices of the bacteria in the black soil and yellow earth did not fluctuate conspicuously, while the Shannon index in red earth decreased significantly after 10 and 14 days of treatment, and the Simpson index increased after 7, 10 and 14 days of stress. Moreover, the Chao1 and Ace indices did not prominently change in the black soil or yellow earth, but after 10 and 14 days of stress, the Chao1 and Ace indices decreased significantly in the red earth (Figure 3). Microbial richness and diversity in the black soil did not significantly change after 7, 10 and 14 days of stress. After 7 days of stress, the Shannon and Simpson indices of the yellow earth plants decreased significantly. In red earth, the Shannon and Ace indices increased obviously after 10 and 14 days, respectively. After 7 days of PTC stress, the Shannon index of the red earth products decreased significantly. The four indices all decreased visibly. After 14 days, the Shannon and Chao1 indices still decreased significantly. The Simpson index and ACE index did not dramatically change (Figure S1). After PTC treatment, the abundance of bacterial and fungal microorganisms decreased, but this inhibition was temporary, and the Chao1 index tended to increase after 14 days. These findings indicated that PTC-related stress might affect the abundance and community structure of rhizosphere microorganisms, especially those associated with red earth.

**Figure 4:**
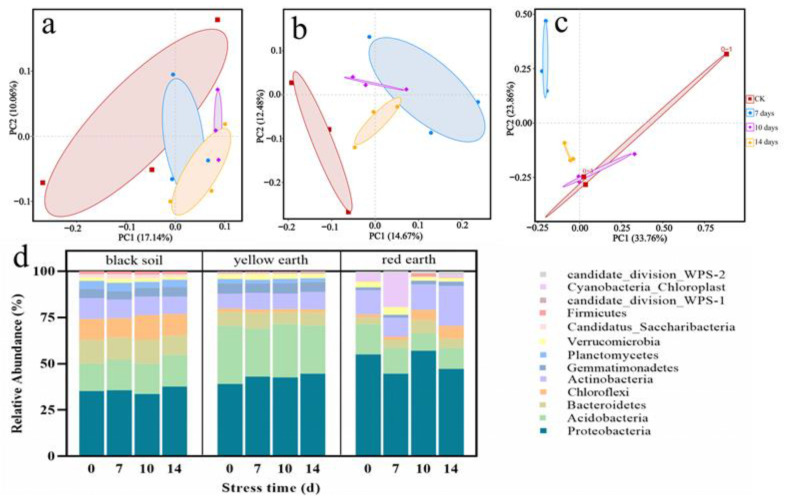
The effect of PTC on rhizosphere bacterial microorganisms in soybean. (**a**–**c**) Principal component analysis (PCA) of the soybean rhizosphere bacterial communities after 7 d, 10 d and 14 d of PTC treatment ((**a**): black soil, (**b**): yellow earth; (**c**): red earth); (**d**) the relative abundance of the top 10 microbes (phylum level) in rhizosphere soil samples after 7 d, 10 d and 14 d of PTC treatment.

At the OTU level, principal component analysis (PCA) was performed based on the Euclidean distance of the separated groups. Principal component 1 (PC1) and principal component 2 (PC2) were the two components that caused the greatest differences in the samples, and the contribution rates were 17.14% and 10.06%, respectively, in the black soil (Figure 4a); 14.67% and 12.48%, respectively, in the yellow soil (Figure 4b); and 33.76% and 23.86%, respectively, in the red earth (Figure 4c). Their contribution rates were 17.14% and 10.06% in the black soil, 14.67% and 12.48% in the yellow earth and 33.76% and 23.86% in the red earth, respectively (Figure S2a,b). The Simpson and Shannon indices combined with the PCA results showed that PTC changed the community structure of the rhizosphere microorganisms. The community structure of the rhizosphere microorganisms changed after PTC treatment, and the specific changes need further analysis.

The effect of PTC on the structure of inter-root microbial communities in three soil types was analyzed in a principal component analysis. An analysis of the microbial communities ranked in the top ten in terms of phylum-level abundance showed that the dominant taxa of bacterial microorganisms in the three soil types were *Proteobacteria* (57.08–33.70% of all phyla), *Actinobacteria* (21.79–7.94% of all phyla), *Bacteroidota* (12.63–3.56% of all phyla) and *Acidobacteriota* (31.36–9.19% of all phyla) (Figure 4d). Thus, these four microorganisms play an important role in the soil. Research has shown that there are groups of beneficial bacteria that contribute to plants growing healthily [34,35]. For example, they can aid in the growth of leguminous root nodules and enhance the absorption of nutrients by plants [36]. *Proteobacteria* and *Acidobacteriota* are two important plant flora, and their continuous enrichment in the plant rhizosphere helps plants to resist stress [37]. For example, the typical actinomyces *Nocardioidaceae* is strongly associated with increases in stem biomass and heavy metal uptake during super accumulating plant growth in soil [38]. The relative abundance of *Sphingomonadaceae* in the *Proteobacteria* increased after PTC stress, which also confirmed this finding (Figure S3). *Sphingomonadaceae* is a new microbial resource with strong metabolic capacity [39]. In general, plants subjected to stress may attract some beneficial microorganisms to resist stress through the rhizosphere. Not only that, but plants defend themselves against pathogen attacks by recruiting beneficial bacteria to the root zone and passing on their legacy to the next generation [40].

The top ten fungi according to relative abundance at the phylum level were selected (Figure S2d), and the dominant flora were *Ascomycota* (45.92–87.95% of all phyla) and *Mortierellomycota* (4.53–39.47% of all phyla). After PTC stress, the relative abundance of fungi in different phyla in black soil did not change significantly. After PTC treatment, the relative abundance of *Ascomyeota* decreased significantly, especially in the yellow earth and red earth. The abundance of *Mortierellomycota* and the specific flora *Chytridiomycota* also decreased visibly after 10 days of stress in the red earth. The dominance of soil fungi differs among different soils, and each soil has its own unique soil microbial community structure [41].

At the family level, the abundances of *Ascodesmidaceae, Helotiaceae* and *Sordariales_fam_Incertae_sedis* which had high abundances, increased significantly after stress in the yellow earth. The three fungi all belong to the phylum *Ascomycota*. The fungi with clear relative abundance changes in the red earth were *Mortierellaceae* and *Helotiaceae*. The abundance of *Mortierellaceae* in the red earth species decreased dramatically after 10 days of stress, while that in *Helotiaceae* increased significantly (Figure S4). The analysis showed that the fungal microbial communities in the different soil types were different. This difference is mainly related to differences in soil nutrients and soil physical and chemical properties [42]. In summary, the difference analysis of fungal dominant flora in different soil types indicated that PTC stress can affect dominant soil fungal flora and change the soil microbial community structure, and there are differences in the bacterial community structures among different soil types. Some studies have also shown significant effects of pesticide use on inter-root fungal and bacterial communities in maize and soybean [43]. Thus, there may be significant and unintended effects on non-target organisms.

At the level of the bacterial microbiome, the abundance of microorganisms with a low relative abundance changed more clearly than of the abundance of those the ordinary groups (Figure 3). The abundance of bacteria and microorganisms plays an important role in plant growth [36]. In some cases, some bacterial communities contribute to nutrient cycling in plants, helping plants to resist stress and respond quickly to environmental changes for healthy plant growth. Understanding microbial communities and plant stress response mechanisms can help to better manage plants and reduce pesticide use [40].

### 3.4. PICRUSt Functional Prediction

Dominant bacteria are critical in maintaining soil ecological functions. We used PICRUSt analysis for the predicted gene abundance within metabolic pathways based on high-throughput sequencing data. The COG function prediction of bacterial community was obtained by comparing it with the COG database (Figures S5a–S7a). There was no significant difference in the functional abundance of COG in bacterial communities after 7, 10 and 14 days of PTC stress compared with the control group in these three different types of soil. In each group, the amounts of amino acid transport and metabolism were higher. The function of the KEGG pathway in the rhizosphere bacterial communities was mainly carried out through six pathways in the three different soil types (Figures S5b–S7b), which are metabolic, genetic information processing, environmental information processing, cellular processes, organismal systems and human diseases, respectively. The changes in the KEGG pathway were discrepant after PTC stress in different soil types. In the black soil, the relative prevalence of amino acid metabolism, carbohydrate metabolism and membrane transport pathway was decreased after 10 days of PTC stress (Figure S5b). After 7 days of PTC stress, the relative prevalence of amino acid metabolism, carbohydrate metabolism and the membrane transport pathway was reduced in the yellow earth (Figure S6b). In the red earth, metabolic pathways and genetic information processing pathways are more abundant. After 7 and 10 days of PTC stress, the relative amounts of amino acid metabolism, carbohydrate metabolism, energy metabolism, translation, and replication and repair pathway, and membrane transport pathway was diminished (Figure S7b). There was no significant change in the relative abundance of cellular processes, organismal systems and human diseases pathways in the three different soil types.

Amino acids are the building blocks of proteins and play a central role in many other physiological processes in plants. In addition, secondary metabolites of amino acid formation have many important functions in plants, such as signaling, defense, interaction with other organisms and light protection [44]. The lower metabolism-related functions may be closely related to the stress response of soybean plants. Membrane transport regulates the passage of solutes such as ions and small molecules through biofilms to promote the rapid development of bacterial growth [45]. The inhibition of membrane transport pathways may affect the number and diversity of bacterial microorganisms. The compensatory function of the inter-root microbiota in helping plants to withstand pesticide stress has been demonstrated [46]. Stressed plants recruit beneficial bacterial communities by increasing the release of primary metabolites (e.g., amino acids, fatty acids, and lysophosphatidylcholine) from root secretions. When pesticides are applied at low doses, the microbial compensatory effect overcomes the pesticide stress and thus promotes plant growth. However, at high doses of pesticides, the microbial compensatory effect was not sufficient to counteract the pesticide stress, resulting in inhibition of plant growth.

### 3.5. Relationships between the Microbial Community and Soil Enzyme Activity and Soil Properties

The Spearman correlation analysis revealed that the rhizosphere microbial community structure (MBM, F/N) was significantly correlated with basic soil physicochemical properties (pH, SOC, TOC, TN, TP) and soil enzyme activity (CAT, DHA, SC, NP, β-GC) (*p* < 0.05; Figures S8–S10), MBM and F/B were negatively correlated with TP and positively correlated with other soil physicochemical properties. The positive correlation between MBM and SOC is typical [47], and our conclusions are consistent with these findings (Figures S8–S10). CAT can promote the decomposition of hydrogen peroxide, which is beneficial for preventing the toxic effects of hydrogen peroxide on organisms and is associated with the soil organic matter content and microorganism quantity [23,25]. CAT activity was negatively correlated with MBM and SOC in the three soils (Figures S8a–S10a). After PTC stress, the CAT activity in all three soils increased, indicating that hydrogen peroxide was produced in the rhizosphere and may have had a toxic effect on the rhizosphere, thus reducing the content of soil organic matter and the number of microorganisms. The products of SC are closely connected to the content of nutrients (such as phosphorus) in soil, the number of microorganisms and the intensity of soil respiration [48]. In black soil, NP and TP are negatively correlated with MBM, while in yellow earth and red earth, NP and TP are positively correlated with MBM. This condition is related to the pH of the soil itself [49]. In an ecosystem with limited phosphorus, plants compete with microorganisms, and acidic soil phosphorus is mainly bound to metal oxides, making it more readily available to microorganisms than to plants [50]. DHA reflects the overall activity of microbial metabolism, while SC is closely related to soil respiration intensity and soil fertility and can be used as an important index for evaluating soil fertility [51,52]. The SOM content was positively correlated with SC activity (Figures S8a–S10a). DHA activity increased and SC activity reduced (Figure 2b,e), indicating that the metabolic activity of rhizosphere microorganisms and soil respiration intensity declined after PTC stress. These two parameters were negatively correlated (Figures S8a–S10a). These results indicated that the metabolic activity of rhizosphere microorganisms and soil respiration intensity decreased after PTC stress.

The relationships between UE activity and organic carbon are not identical and include negative correlations [34], positive correlations [12] and no correlations [53]. This difference is associated with the stability of UE itself, and its stability is related to the soil’s own conditions.

Fungal communities had less of an effect on soil variables than bacterial communities. In addition to soil enzyme activity and pH, fungal communities were not strongly correlated with rhizosphere organic carbon or nutrients (Figures S8c,d–S10c,d). The results showed that the fungal community played a lower role in resistance to PTC stress than the bacterial community. Therefore, compared with the fungal community, the bacterial community has greater potential to help soybean plants resist PTC stress. Changes in these key fungal groups induced by PTC stress intensely affected key bacterial groups (Figures S8b–S10b), suggesting that these key fungal groups may indirectly assist soybean growth under PTC stress by affecting bacterial communities. Some fungal communities have direct effects on the community structure of key bacterial microorganisms and indirect effects on helping plants resist stress and aiding plant growth. These findings pave the way for designing improved pesticide application strategies and contribute to a better understanding of how the microbiota can be used as an eco-friendly method to mitigate chemically induced stresses in crops.

## 4. Conclusions

This study revealed changes in enzyme activity and microbial community structure in the rhizosphere soil of soybean plants after PTC treatment. It was confirmed that PTC stress in soil significantly induced oxidative stress, which had an effect on plant growth. Importantly, PTC significantly altered the rhizosphere microbial composition, community structure and function of soybean plants. The changes in microbial community structure in different types of soil are not the same and are closely related to the physical and chemical properties of the soil itself. The abundance of metabolism-related and membrane transport pathways decreased after PTC stress, which may have changed the community structure of rhizosphere bacteria. There are still significant limitations to predicting the function of related bacteria by PICRUSt alone. A correlation analysis of soil physical and chemical properties, enzyme activities and rhizosphere microorganisms with high abundance ratios revealed that changes in physical and chemical properties and soil enzyme activities affect rhizosphere microbial activities and lead to changes in microbial community structure. The results obtained in this study indicate that residual PTC in soil may affect the growth of soybean plants by affecting the enzyme activity and microbial community structure of the rhizosphere soil. Therefore, farmers or environmental regulators should improve pesticide utilization, increase control efficiency, and reduce soil contamination, thereby reducing the impact on soil microbial and enzyme activity. However, the factors driving the changes in inter-root microorganisms as well as the deeper biochemical mechanisms in the soybean inter-root have not been investigated at a deeper level. Therefore, in future studies, we will further investigate the interactions between soil physicochemical properties and soil enzyme activities, combined with metabolomics to reveal the deeper causes of inter-root community changes. At the same time, the biochemical mechanisms of amino acid and carbohydrate metabolism and the inhibition of membrane transport pathways will be further investigated; after that, the main microorganisms and metabolites will be screened and validated in in vitro experiments will be carried out to determine the definitive mechanisms of microbial changes. The way that PTC altered the microbial composition of the rhizosphere and the community structure is particularly concerning.

## Figures and Tables

**Table 1:** Basic physical and chemical properties of the soil.

Soil	pH	SOC (g/kg)	TOC (g/kg)	TP (g/kg)	TK (g/kg)	CEC (cmol)	Sand (%)	Silt (%)	Clay (%)
black soil	7.66	13.42	51.65	0.60	13.42	43.44	37.69	32.74	29.57
yellow earth	8.32	2.83	5.31	0.15	11.58	39.66	20.12	57.52	22.46
red earth	4.98	6.36	6.40	0.11	15.69	28.05	18.73	18.93	62.34

SOC: soil organic matter; TOC: soil organic carbon; TP: total soil potassium; TK: total soil potassium; CEC: soil cation exchange capacity.

## Data Availability

The original contributions presented in the study are included in the article/Supplementary Material, further inquiries can be directed to the corresponding author.

## Supplementary Materials

The following supporting information can be downloaded at: https://www.mdpi.com/article/10.3390/toxics12100692/s1, Figure S1: The changes of diversity indices (Simpson, Shannon) and richness (Chao1, Ace) of soybean rhizosphere fungal microorganism community after 7 d, 10 d and 14 d PTC treatment. * Correlation is significant at *p* < 0.05 (two-tailed); ** Correlation is significant at *p* < 0.01 (two-tailed); *** Correlation is significant at *p* < 0.001 (two-tailed). Figure S2: The effect of PTC on rhizosphere fungal microorganisms of soybean. (a–c) Principal component analysis (PCA) of the soybean rhizosphere bacterial communities after 7 d, 10 d and 14 d of PTC treatment (a: black soil, b: yellow earth; c: red earth); (D) The relative abundance of the top 10 microbes (Phylum level) in rhizosphere soil samples after 7 d, 10 d and 14 d of PTC treatment. Figure S3: The effects of prothioconazole on the abundances of bacteria in soil after its application to soybean plants. Heatmap showing the change in the relative abundance of the top 20 microbes (at the family level) in rhizosphere soil samples after 7, 10 and 14 days of PTC treatment (a: black soil; b: yellow earth; c: red earth). Figure S4: Effects of prothioconazole on the abundances of fungi in soil after the application on soybean. Heatmap showing the change of relative abundance of the top 20 microbes (Family level) in rhizosphere soil samples after 7 d, 10 d and 14 d of PTC treatment. (a: black soil; b: yellow earth; c: red earth). Figure S5: Functional changes of rhizosphere bacterial microbial communities in black soil after PTC stress (a: COG functional abundance; b: KEGG functional abundance). Figure S6: Functional changes of rhizosphere bacterial microbial communities in yellow earth after PTC stress. (a: COG functional abundance; b: KEGG functional abundance). Figure S7: Functional changes of rhizosphere bacterial microbial communities in red earth after PTC stress. (a: COG functional abundance; b: KEGG functional abundance). Figure S8: Spearman’s correlation of (a) the soil microbial community, enzyme activities, and soil properties after incubation in black soil and relationships between bacterial taxa and fungal taxa (phylum level) (b). Correlation heatmap of bacterial taxa (phylum level) (c) and fungal taxa (phylum level, d) with soil properties. The soil properties included pH, SOC, soil organic carbon content, TOC, total organic carbon, TN, total nitrogen content, and TP, total phosphorus content. MBM, total microbial biomass; F/B, ratio of fungi to bacteria. * Correlation is significant at *p* < 0.05 (two-tailed); ** correlation is significant at *p* < 0.01 (two-tailed); *** correlation is significant at *p* < 0.001 (two-tailed). Figure S9: Spearman’s correlation of (a) soil microbial community, enzyme activities, and soil properties after incubation in yellow earth, and relationships between bacterial taxa and fungal taxa (phylum level). (b). Correlation heat map of bacterial taxa (phylum level, c) and fungal taxa (phylum level, d) with soil properties. Soil properties include pH, SOC, soil organic carbon content; TOC, total organic carbon; TN, total nitrogen content; TP, total phosphorus content. MBM, total microbial biomass; F/B, ratio of fungi to bacteria. * Correlation is significant at *p* < 0.05 (two-tailed); ** Correlation is significant at *p* < 0.01 (two-tailed); *** Correlation is significant at *p* < 0.001 (two-tailed). Figure S10: Spearman’s correlation of (a) soil microbial community, enzyme activities, and soil properties after incubation in red earth, and relationships between bacterial taxa and fungal taxa (phylum level). (b). Correlation heat map of bacterial taxa (phylum level, c) and fungal taxa (phylum level, d) with soil properties. Soil properties include pH, SOC, soil organic carbon content; TOC, total organic carbon; TN, total nitrogen content; TP, total phosphorus content. MBM, total microbial biomass; F/B, ratio of fungi to bacteria. * Correlation is significant at *p* < 0.05 (two-tailed); ** Correlation is significant at *p* < 0.01 (two-tailed); *** Correlation is significant at *p* < 0.001 (two-tailed).
