# Supplementary material for: Prothioconazole Stress Reduces Bacterial Richness and Alters Enzyme Activity in Soybean Rhizosphere
Source: Toxics. 2024 Sep 25;12(10):692. doi: 10.3390/toxics12100692 (PMC11510772; doi:10.3390/toxics12100692)
Supplement: Supplementary file 1 [file toxics-12-00692-s001.zip › toxics-3215688-SI.pdf]

Supporting Information

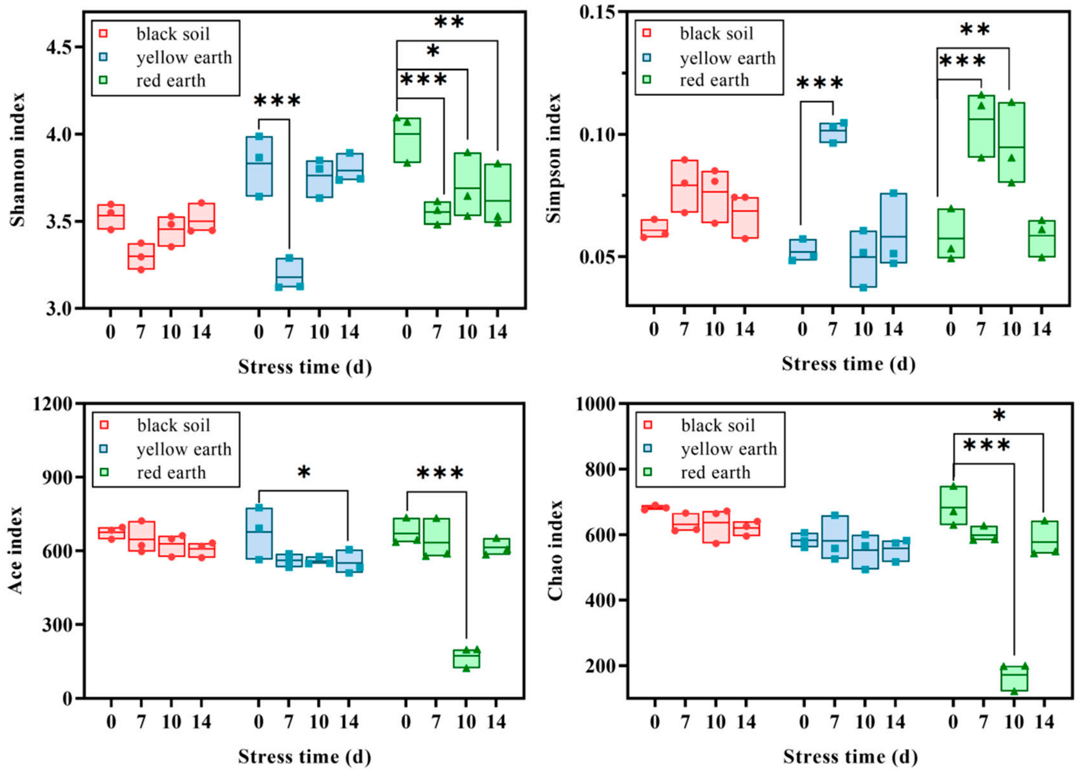

Fig. S1 The changes of diversity indices (Simpson, Shannon) and richness (Chao1, Ace) of soybean rhizosphere fungal microorganism community after 7 d, 10 d and 14 d PTC treatment. * Correlation is significant at P < 0.05 (two-tailed); ** Correlation is significant at P < 0.01 (two-tailed); *** Correlation is significant at P < 0.001 (two-tailed).

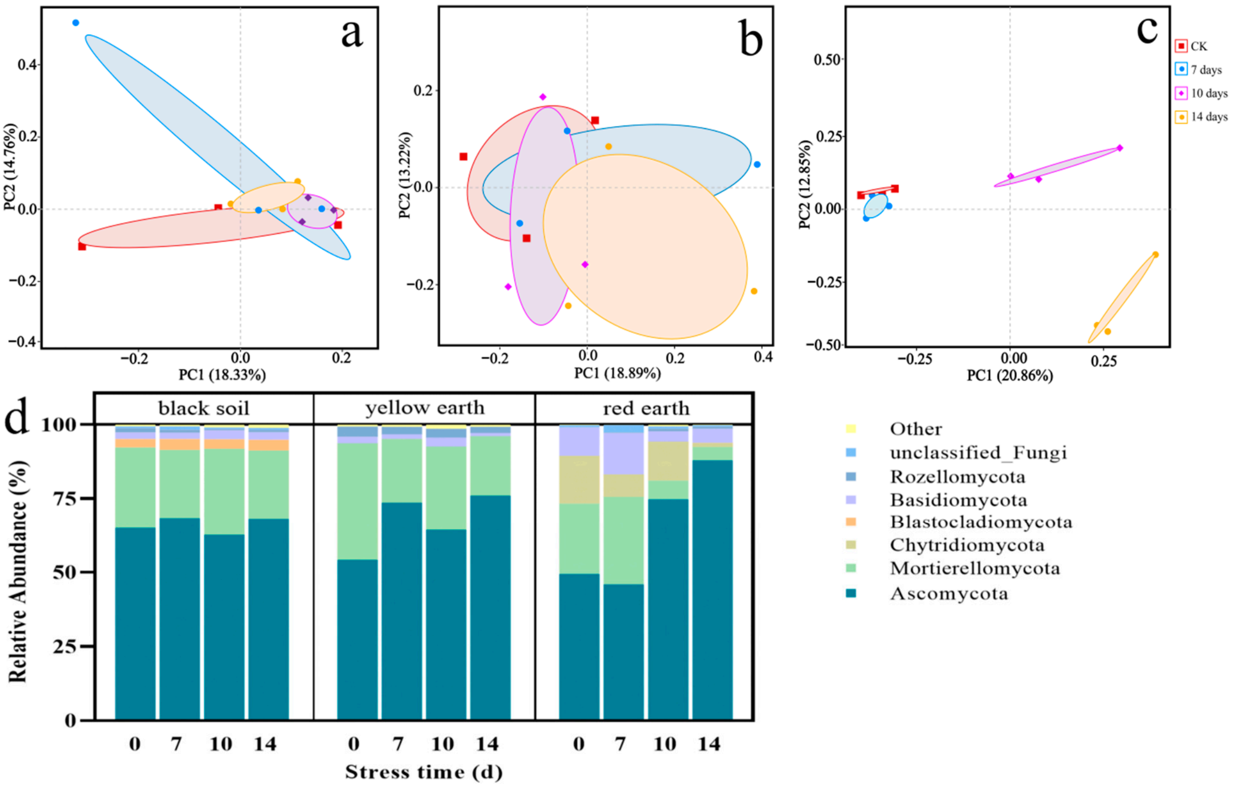

Fig. S2 The effect of PTC on rhizosphere fungal microorganisms of soybean. (a) (b) (c) Principal component analysis (PCA) of the soybean rhizosphere bacterial communities after 7 d, 10 d and 14 d of PTC treatment (a: black soil, b: yellow earth; c: red earth); (D) The relative abundance of the top 10 microbes (Phylum level) in rhizosphere soil samples after 7 d, 10 d and 14 d of PTC treatment.

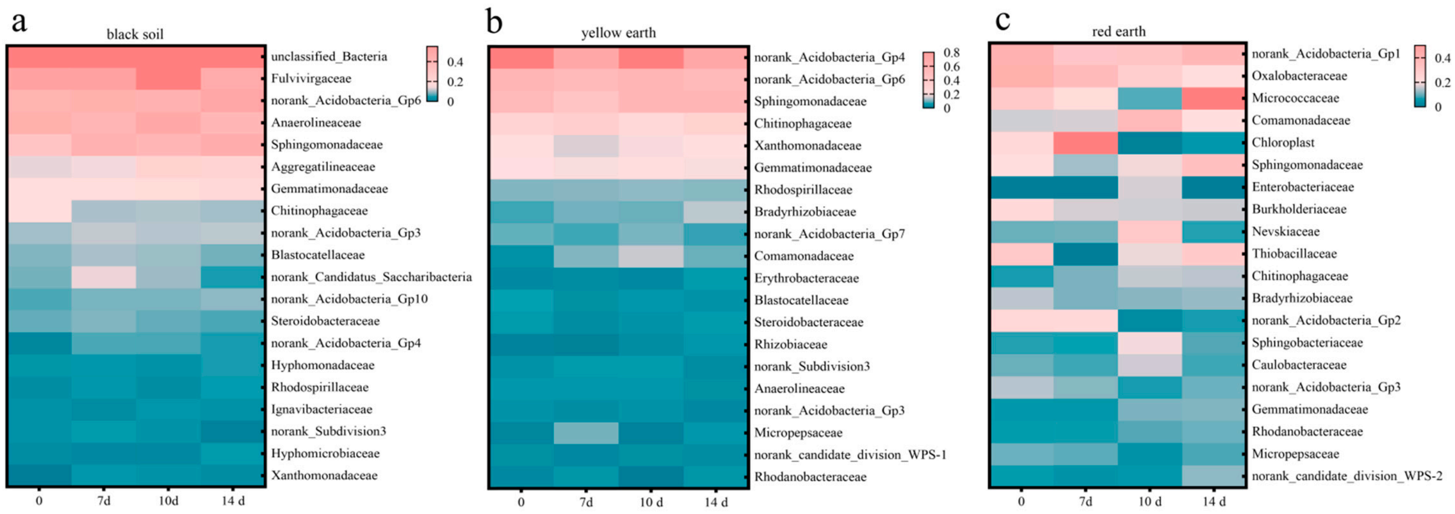

Fig. S3 The effects of prothioconazole on the abundances of bacteria in soil after its application to soybean plants. Heatmap showing the change in the relative abundance of the top 20 microbes (at the family level) in rhizosphere soil samples after 7, 10 and 14 days of PTC treatment (a: black soil; b: yellow earth; c: red earth).

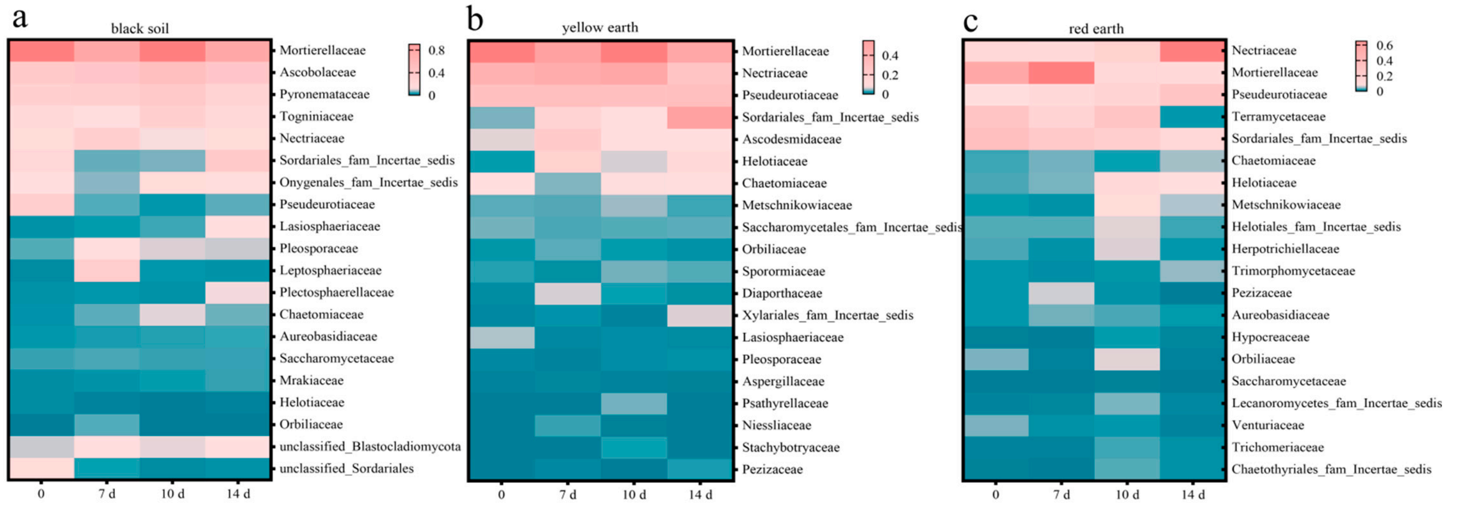

Fig. S4 Effects of prothioconazole on the abundances of fungi in soil after the application on soybean. Heatmap showing the change of relative abundance of the top 20 microbes (Family level) in rhizosphere soil samples after 7 d, 10 d and 14 d of PTC treatment. (a: black soil; b: yellow earth; c: red earth).

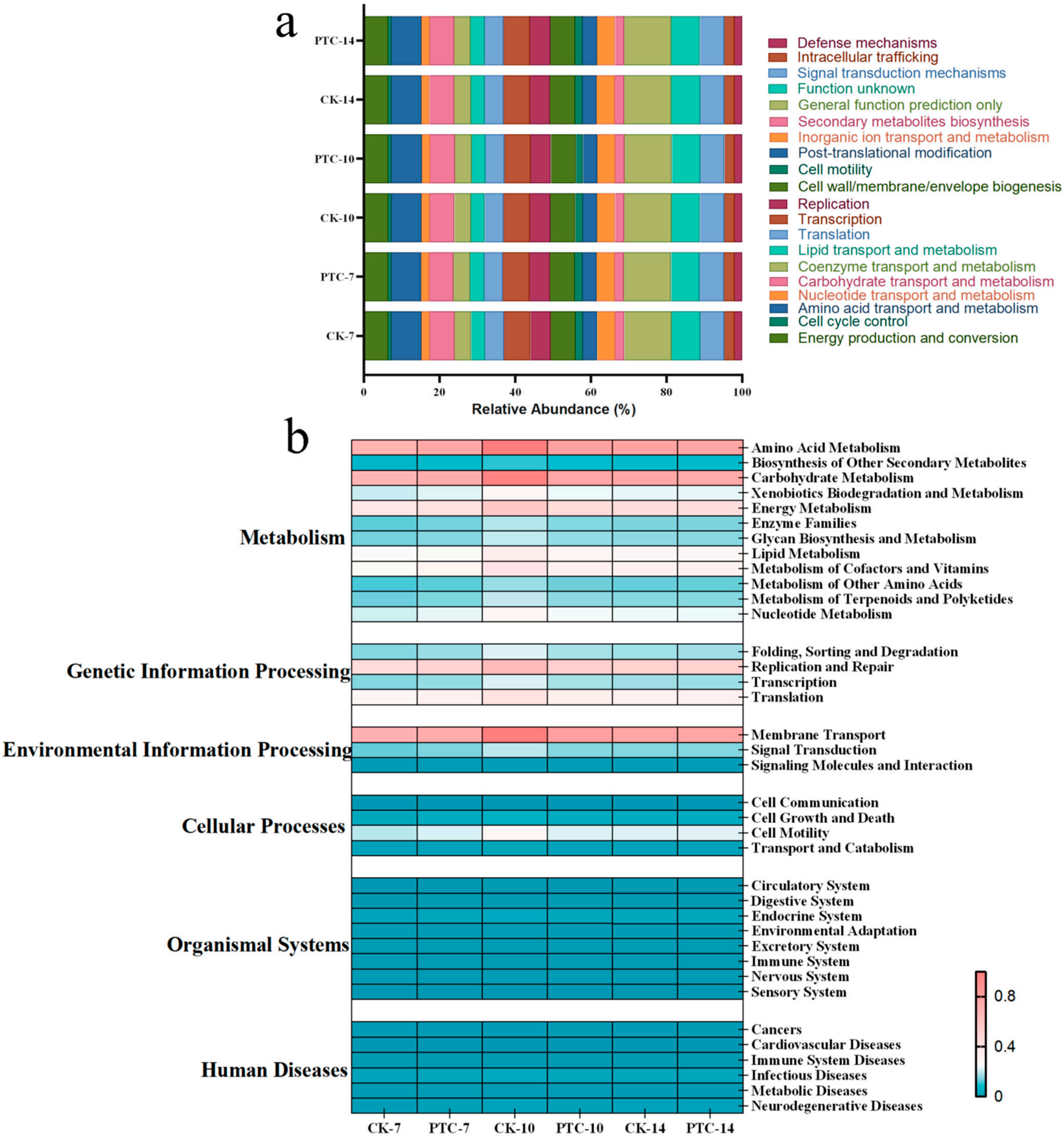

Fig. S5 Functional changes of rhizosphere bacterial microbial communities in black soil after PTC stress (a: COG functional abundance; b: KEGG functional abundance).

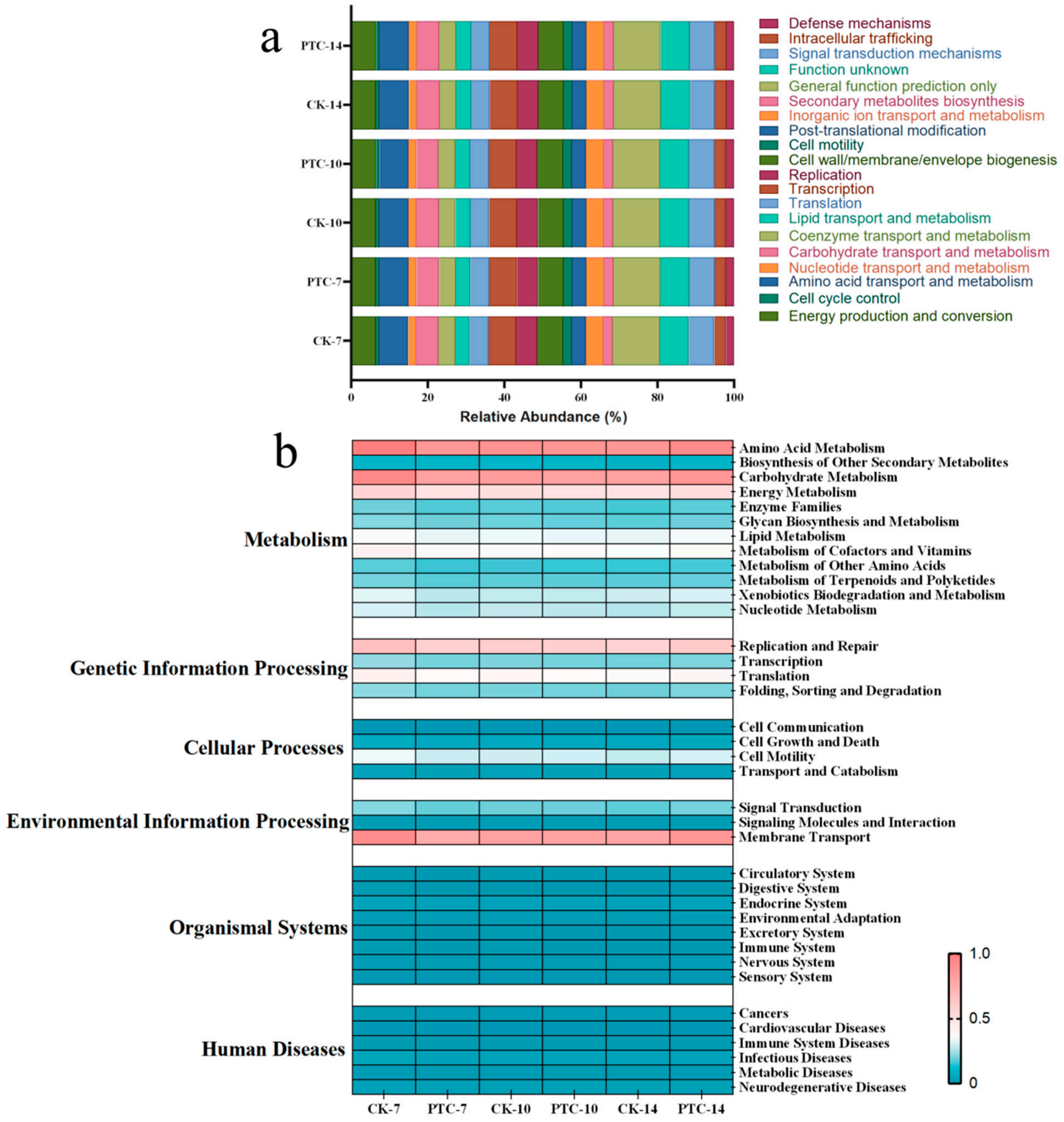

Fig. S6 Functional changes of rhizosphere bacterial microbial communities in yellow earth after PTC stress. (a: COG functional abundance; b: KEGG functional abundance).

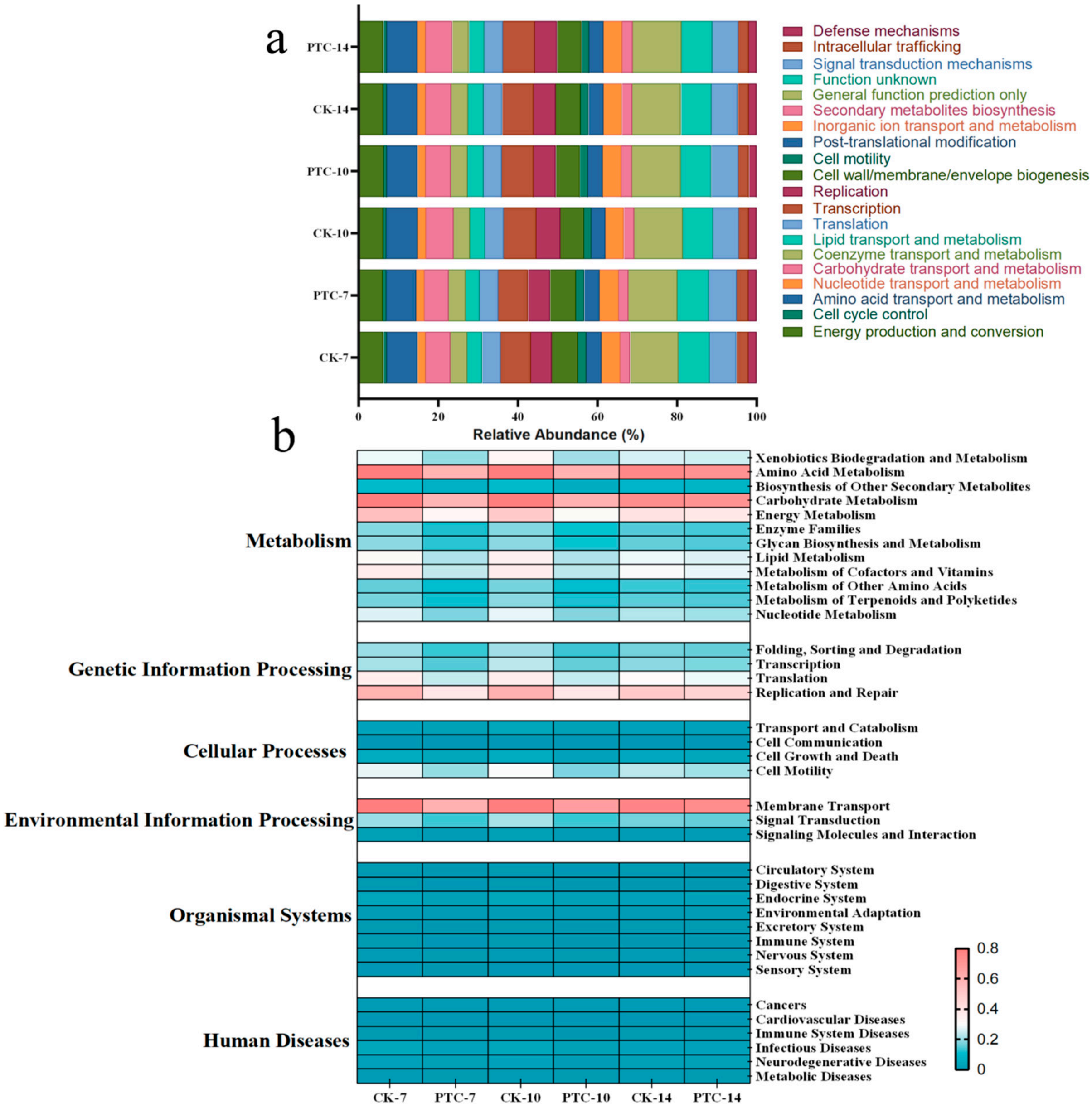

Fig. S7 Functional changes of rhizosphere bacterial microbial communities in red earth after PTC stress. (a: COG functional abundance; b: KEGG functional abundance).

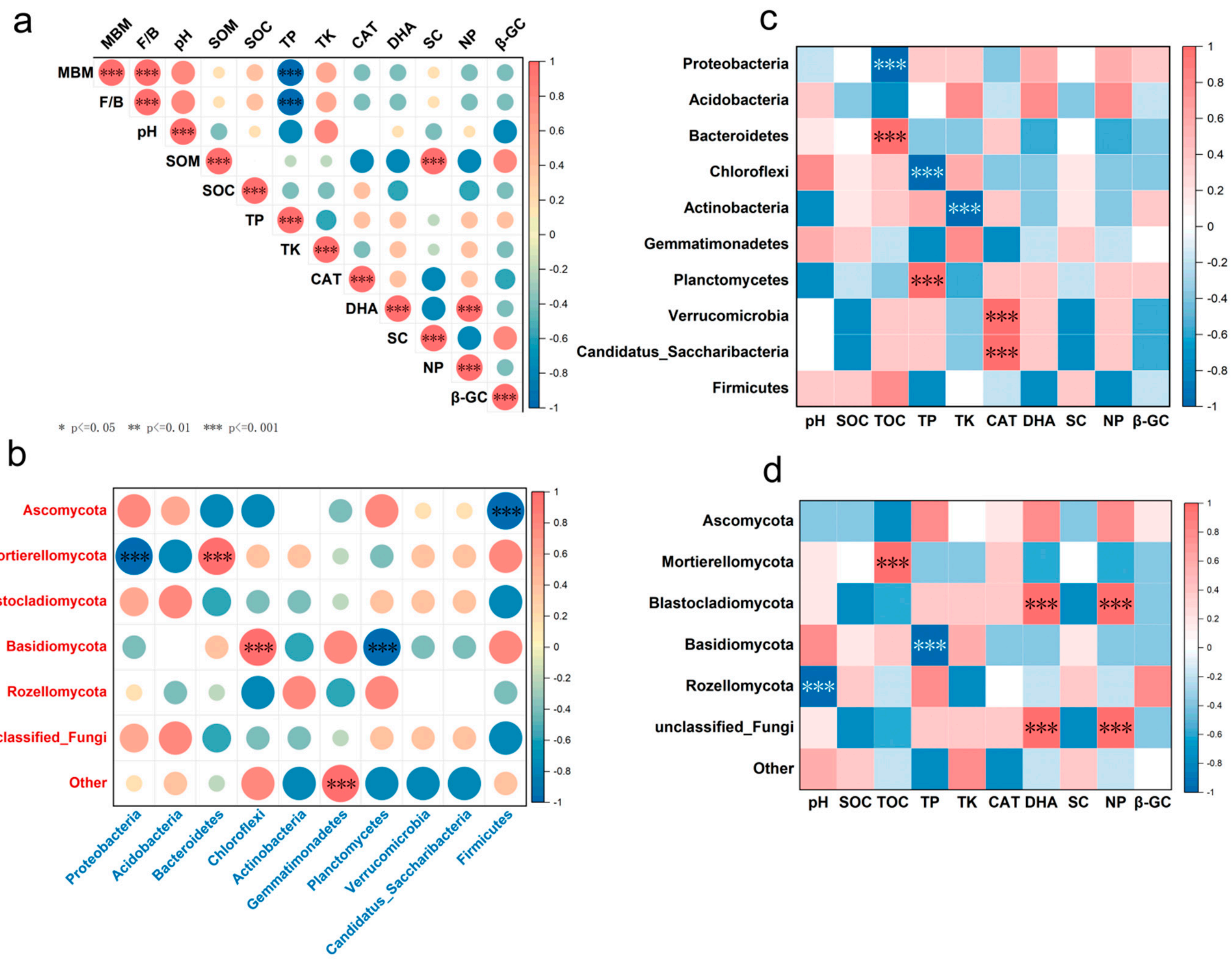

Fig. S8 Spearman’s correlation of (a) the soil microbial community, enzyme activities, and soil properties after incubation in black soil and relationships between bacterial taxa and fungal taxa (phylum level) (b). Correlation heatmap of bacterial taxa (phylum level) (c) and fungal taxa (phylum level, d) with soil properties. The soil properties included pH, SOC, soil organic carbon content, TOC, total organic carbon, TN, total nitrogen content, and TP, total phosphorus content. MBM, total microbial biomass; F/B, ratio of fungi to bacteria. * Correlation is significant at P < 0.05 (two-tailed); ** correlation is significant at P < 0.01 (two-tailed); *** correlation is significant at P < 0.001 (two-tailed).

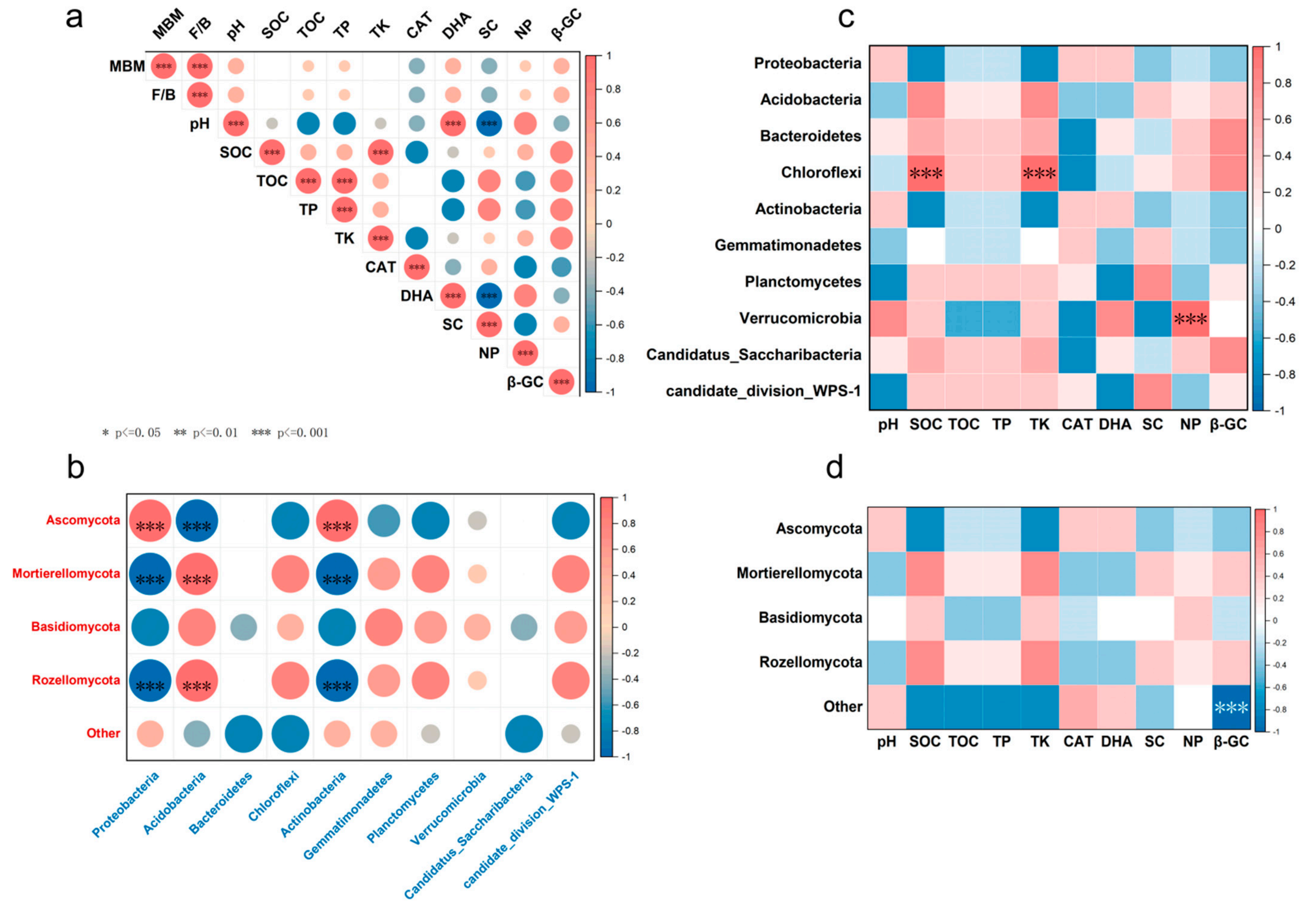

Fig. S9 Spearman's correlation of (a) soil microbial community, enzyme activities, and soil properties after incubation in yellow earth, and relationships between bacterial taxa and fungal taxa (phylum level). (b). Correlation heat map of bacterial taxa (phylum level, c) and fungal taxa (phylum level, d) with soil properties. Soil properties include pH, SOC, soil organic carbon content; TOC, total organic carbon; TN, total nitrogen content; TP, total phosphorus content. MBM, total microbial biomass; F/B, ratio of fungi to bacteria. * Correlation is significant at $P < 0.05$ (two-tailed); ** Correlation is significant at $P < 0.01$ (two-tailed); *** Correlation is significant at $P < 0.001$ (two-tailed).

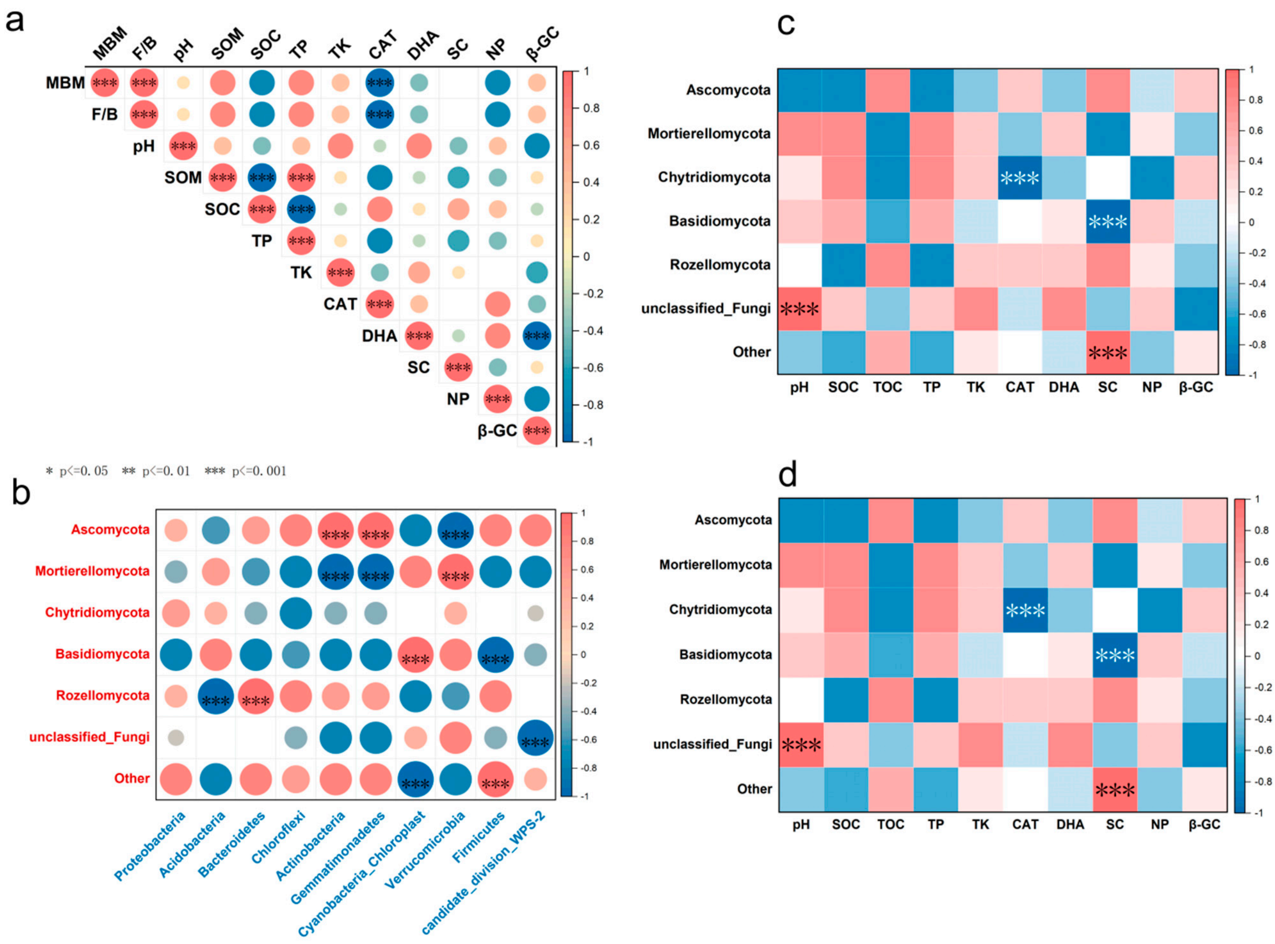

Fig. S10 Spearman's correlation of (a) soil microbial community, enzyme activities, and soil properties after incubation in red earth, and relationships between bacterial taxa and fungal taxa (phylum level). (b). Correlation heat map of bacterial taxa (phylum level, c) and fungal taxa (phylum level, d) with soil properties. Soil properties include pH, SOC, soil organic carbon content; TOC, total organic carbon; TN, total nitrogen content; TP, total phosphorus content. MBM, total microbial biomass; F/B, ratio of fungi to bacteria. * Correlation is significant at P < 0.05 (two-tailed); ** Correlation is significant at P < 0.01 (two-tailed); *** Correlation is significant at P < 0.001 (two-tailed).
